# Molecular subtypes, prognostic and immunotherapeutic relevant gene signatures mediated by DNA methylation regulators in hepatocellular carcinoma

**DOI:** 10.18632/aging.204155

**Published:** 2022-06-30

**Authors:** Rongfeng Shi, Hui Zhao, Suming Zhao, Hongxin Yuan

**Affiliations:** 1 Department of Interventional Radiology, Affiliated Hospital of Nantong University, Nantong 226001, Jiangsu, P.R. China

**Keywords:** DNA methylation, immunotherapy, prognosis, biomarker, hepatocellular carcinoma

## Abstract

Growing evidence has revealed the crucial role of epigenetics in tumor progression and immune response. However, the molecular subtypes and their microenvironment characterization mediated by DNA methylation regulators in hepatocellular carcinoma remain little known. In this study, we comprehensively integrated the transcriptome profiling of twenty DNA methylation regulators in hepatocellular carcinoma. Consensus clustering was used to identify distinct methylation regulator-related molecular subtypes. The prognostic DMS signature was constructed using principal components analysis. Most regulators experienced a low genomic variation, but we found a remarkably difference in mRNA expression of these regulators between normal and tumor tissues. Three distinct methylation regulator-related molecular subtypes were successfully identified according to the expression of 20 regulators, which had substantially different biological characteristics and prognosis. The classic carcinogenic pathways and stromal activity including TGF-beta, p53 and WNT signaling pathway were significantly activated in subtype B, leading to a survival inferiority in subtype B compared to other two subtypes. Further analysis demonstrated the constructed DMS signature was an independent predictive biomarker in patient prognosis. Two anti-checkpoint immunotherapy cohorts demonstrated patients with high DMS presented significantly improved treatment advantages and enhanced responses especially the survival prolonged. Generally, the high DMS groups improved more than 15% clinical response to immunotherapy than low DMS groups. In conclusion, this study identified three DNA methylation regulator-related subtypes with distinct clinical, molecular and biological characteristics, and constructed a prognostic and immunotherapeutic relevant gene signature. It might help to promote individualized immunotherapy for hepatocellular carcinoma from the perspective DNA methylation regulators.

## INTRODUCTION

DNA methylation, as one of the epigenetic modifications most well characterized, was involved in multiple biological processes in mammals. The main form of DNA methylation modification was 5mC, indicating that DNA methylation occurs on the fifth carbon atom of CpG dinucleotide cytosine residues [1–3]. Although technological advancements and novel mechanistic insights have altered strategies for treating hepatocellular carcinoma over the past decade, these improvements can only benefit a small number of patients, with five-year survival rates less than 50% [4–6]. However, a small group of patients with robust responses achieved astounding survival benefits from anti-checkpoint immunotherapy, which was represented by the anti-PD-1/PD-L1 antibodies. Unfortunately, for the great majority of patients, the benefits are either minimal or nonexistent, far from meeting a clinical need. Furthermore, clinical response rates differ both within and between tumor types, indicating there existed intrinsic and adaptive immune resistance to anti-checkpoint immunotherapy [7–9]. It was reported that several genes, regulated by hypomethylation or hypermethylation of the promoter, were significantly correlated with the initiation and progression of hepatocellular carcinoma. Qian et al. reported that the stemness and tumorigenicity in hepatocellular carcinoma were regulated by DNMT1-mediated methylation of BEX1 [10]. Luming et al. revealed that the enforced HOXD3 promoter methylation mediated by MeCP2 was involved in hepatocellular carcinoma progression via HB-EGF/EGFR pathway [11]. However, the molecular subtypes mediated by DNA methylation regulators and their roles in patient survival and immunotherapeutic efficacy remain unknown. In this study, we comprehensively analyzed the genomic characterization of 20 DNA methylation regulators, and their mediated molecular subtypes in hepatocellular carcinoma. We successfully defined three DNA methylation regulator-related molecular subtypes with distinct prognostic features and biological functions in hepatocellular carcinoma based on 369 TCGA samples. We also constructed DNA methylation related signature (DMS) based on the overlapping differentially expressed genes across three subtypes, which was confirmed to be significantly related to the patient's prognosis and efficacy of anti-checkpoint immunotherapy.

## RESULTS

### Genomic characterization of DNA methylation regulators in hepatocellular carcinoma

**Figure 1:**
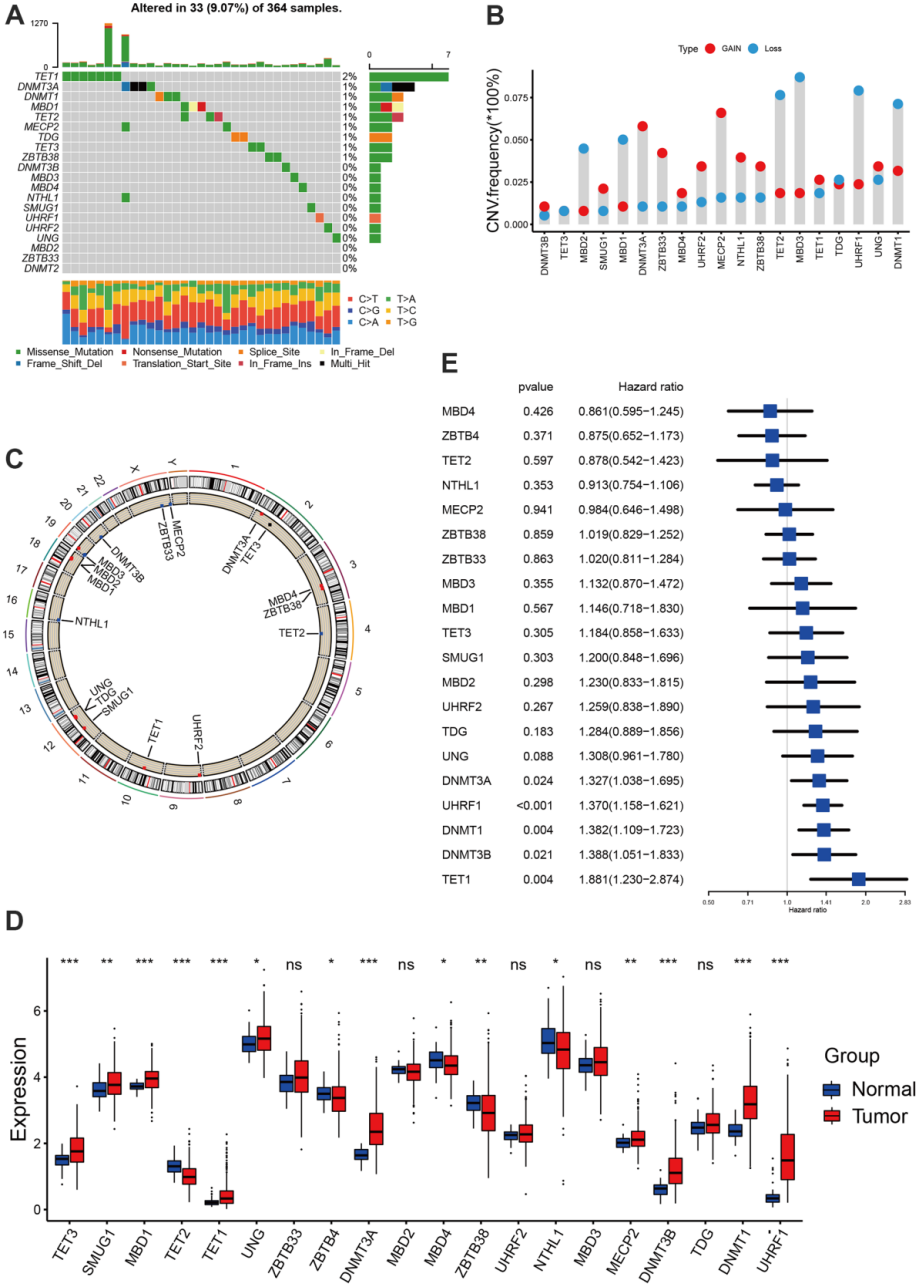
**Landscape of DNA methylation regulators in hepatocellular carcinoma.** (**A**) The mutation landscape of 20 DNA methylation regulators in TCGA-LIHC cohort. (**B**) The Copy number variation frequency of 20 DNA methylation regulators. (**C**) The position of the 20 regulators in the chromosome. (**D**) Expression of 20 regulators in tumor and normal samples based on TCGA-cohort. (**E**) Survival analyses for the 20 regulator genes using univariate Cox regression model. All data analyses were based on the TCGA-LIHC cohort. CNV, copy number variation. Ns, not significant. *P < 0.05; **P < 0.01; ***P < 0.001.

We extracted a total of 20 DNA methylation regulators from TCGA-LIHC and GSE14520 datasets, including 14 readers (SMUG1, NTHL1, TDG, UNG, MECP2, UHRF1, UHRF2, ZBTB4, ZBTB33, ZBTB38, MBD1, MBD2, MBD3, MBD4), 3 erasers (TET1, TET2, TET3) and 3 writers (DNMT1, DNMT3A, DNMT3B) [12]. The analysis of mutational landscape for 20 DNA methylation regulators showed that in hepatocellular carcinoma, only 33 samples of 364 samples experienced at least one mutation, accounting for 9.07% (Figure 1A). The CNV analyses indicated these regulators also a relatively low CNV alteration frequency in hepatocellular carcinoma. TET2, MBD3, UHRF1, DNMT1, MBD2 and MBD2 mainly focused on the frequency of copy number deletion, while DNMT3A, ZBTB33, MECP2 and NTHL1 exhibited copy number amplifications (Figure 1B). The position of 20 regulators in chromosome was shown in Figure 1C. Based on the TCGA-LIHC cohort, we found the expression of DNA methylation regulators between normal tissues and tumor tissues showed significant difference. TET2, ZBTB4, MBD4, ZBTB38 and NTHL1was significantly down-regulated in tumor tissues, while TET3, SMUG1, MBD1, TET1, UNG, DNMT3A, MECP2, DNMT3B, DNMT1 and UHRF1 was significantly up-regulated in tumor tissues (Figure 1D). Survival analyses with the univariate Cox regression model revealed their crucial roles in the patient outcomes, of these, DNMT3A, UHRF1, DNMT1, DNMT3B and TET1 the risk factors for hepatocellular carcinoma. However, other regulators did not show a significant effect on prognosis (Figure 1E). Additionally, we found tumors with the up-regulated eraser genes showed a high expression of writer genes, except for TET2 eraser gene (Supplementary Figure 1A–1C).

### Consensus clustering for identifying methylation regulator-related molecular subtypes

**Figure 2:**
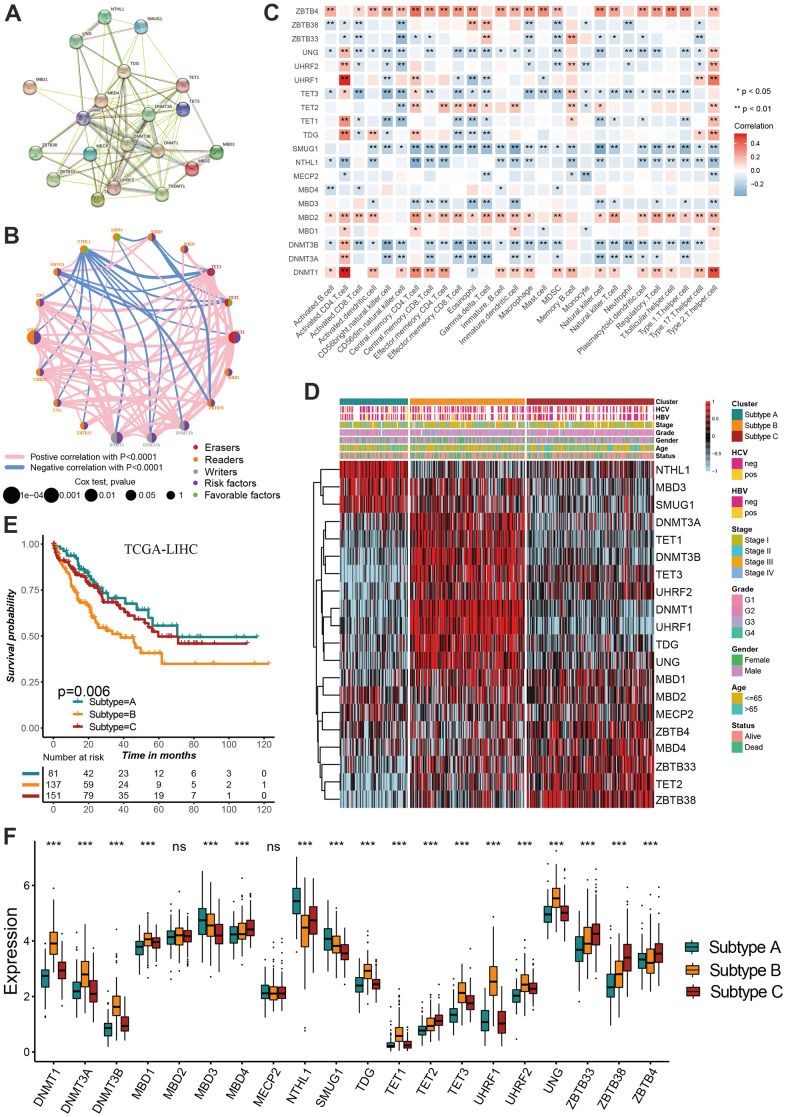
**Identification of DNA methylation regulator-related molecular subtypes in hepatocellular carcinoma.** (**A**) The protein-protein interactions (PPI) network between DNA methylation regulators using STRING database. (**B**) A network was used to visualize the prognostic significance and expression correlation among these regulators. (**C**) The correlation between DNA methylation regulator expression and immune cell infiltration levels. (**D**) The hierarchical clustering of 20 DNA methylation regulators among three molecular subtypes. (**E**) Survival analyses for three distinct DNA methylation regulator-related molecular subtypes. (**F**) DNA methylation regulators expressed in the three molecular subtypes. All data analyses were based on the TCGA-LIHC cohort. Neg, negative; Pos, positive; Ns, not significant. *P < 0.05; **P < 0.01; ***P < 0.001.

Based on the protein-protein interaction (PPI) network constructed by the STRING, we found these regulators showed a widespread protein interaction (Figure 2A). Further analysis showed that these regulators exhibited significant positive correlations at mRNA expression levels (Figure 2B). A network was used to visualize the prognostic significance and interaction among these regulators.(Figure 2B) Previous studies showed that DNA methylation play anti-tumor immune effects by regulating cell infiltration in the tumor microenvironment [13, 14]. We therefore explored the correlation between tumor environment cell infiltration and DNA methylation regulators. We found ZBTB4, MBD2 and DNMT1 present a significant correlation with most cell infiltration abundance (Figure 2C). The consensus clustering was used to identify the distinct molecular subtypes mediated by DNA methylation regulators in hepatocellular carcinoma. According to the expression of 20 regulators, all tumor samples were clustered into three distinct subtypes. We termed these subtypes as methylation regulator-related molecular subtype A, B and C, respectively (Supplementary Figure 2). Survival analysis revealed a significant difference on prognosis between three subtypes, with particularly prominent survival advantage in subtype A and C, and the survival inferiority in subtype B (Figure 2E). NTHL1, MBD3 and SMUG1 were characteristically expressed in subtype A, DNMT3A, TET1, DNMT3B, TET3, UHRF2, DNMT1, UHRF1, TDG and UNG characteristically expressed in subtype B, while ZBTB4, MBD4, ZBTB33, TET2 and ZBTB38 characteristically expressed in subtype C (Figure 2D–2F).

### Characteristics of distinct methylation regulator-related molecular subtypes

**Figure 3:**
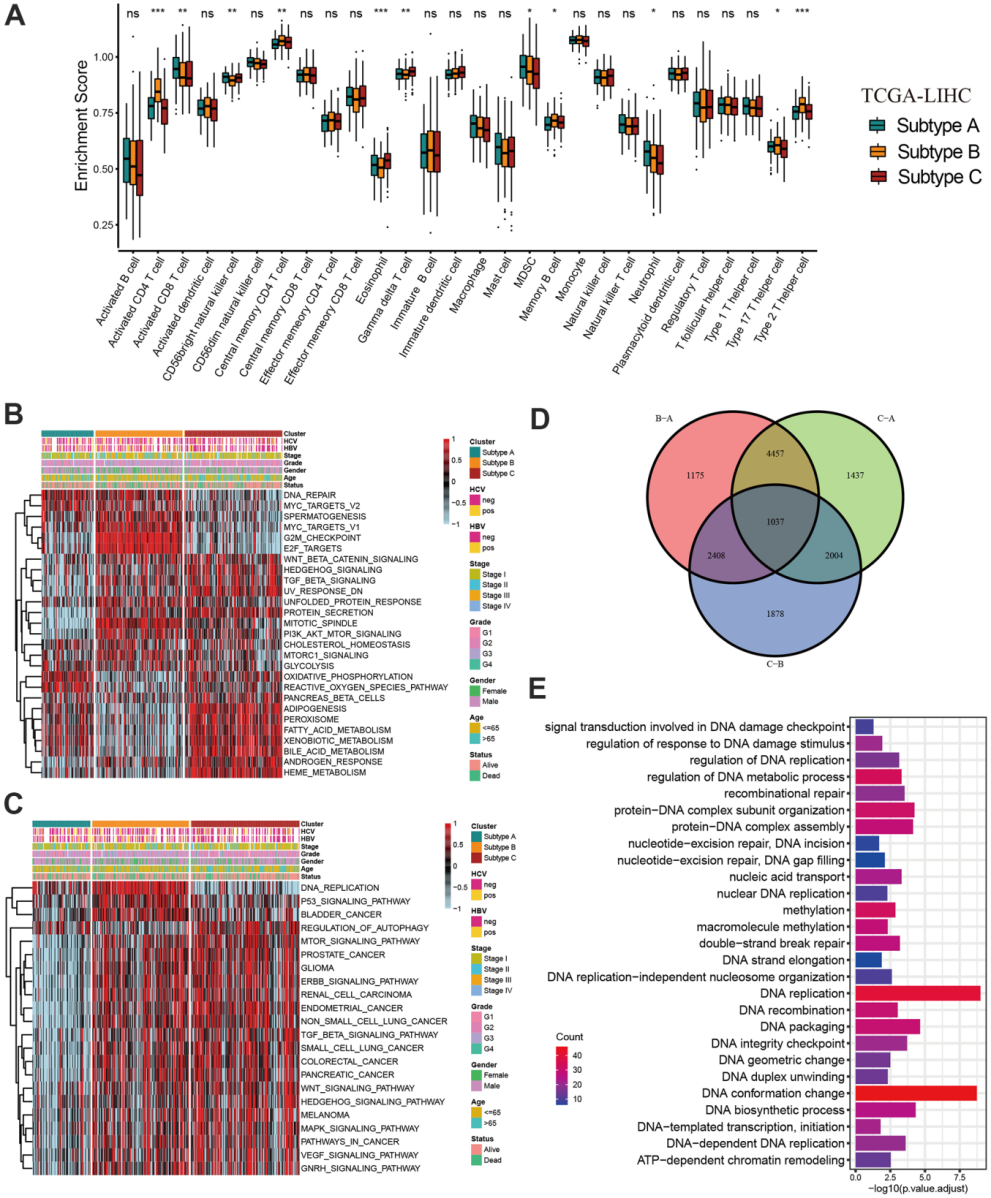
**Characteristics of distinct DNA methylation regulator-related molecular subtypes.** (**A**) The abundance of 28 tumor microenvironment cell infiltration among three molecular subtypes. (**B**, **C**) GSVA enrichment showing the activation states of biological pathways in distinct molecular subtypes. (**D**) The Venn diagram showing 1037 overlapping differentially expressed genes (DEGs) between three DNA methylation regulator-related molecular subtypes. (**E**) GO functional enrichment analyses for 1037 overlapping differentially expressed genes. All data analyses were based on the TCGA-LIHC cohort. Neg, negative; Pos, positive; Ns, not significant. *P < 0.05; **P < 0.01; ***P < 0.001.

Considering the association between tumor microenvironment and DNA methylation, we investigate the difference in immune infiltration abundance among three subtypes. The infiltration level of activated CD8 T cell, activated CD4 T cell, CD56bright natural killer cell, gamma delta T cell, neutrophil and Type 2 T helper cell were significantly different between the three molecular subtypes (Figure 3A). Although subtype B showed a relatively immune cell infiltration, numerous classic carcinogenic pathways and stromal activity were significantly activated in subtype B including WNT signaling pathway, p53 signaling pathway and TGF-beta signaling pathway, leading to a survival inferiority compared to other two subtypes (Figure 3B, 3C). The transcriptome difference between three subtypes was investigated to further reveal the underlying biological characteristics in there three molecular subtypes, and a total of 1037 overlapping differentially expressed genes (DEGs) were obtained (Figure 3D). The GO enrichment analysis demonstrated the DEGs were prominently associated with the DNA methylation related biological pathways (Figure 3E).

### Construction of prognostic signature based on overlapping DEGs

**Figure 4:**
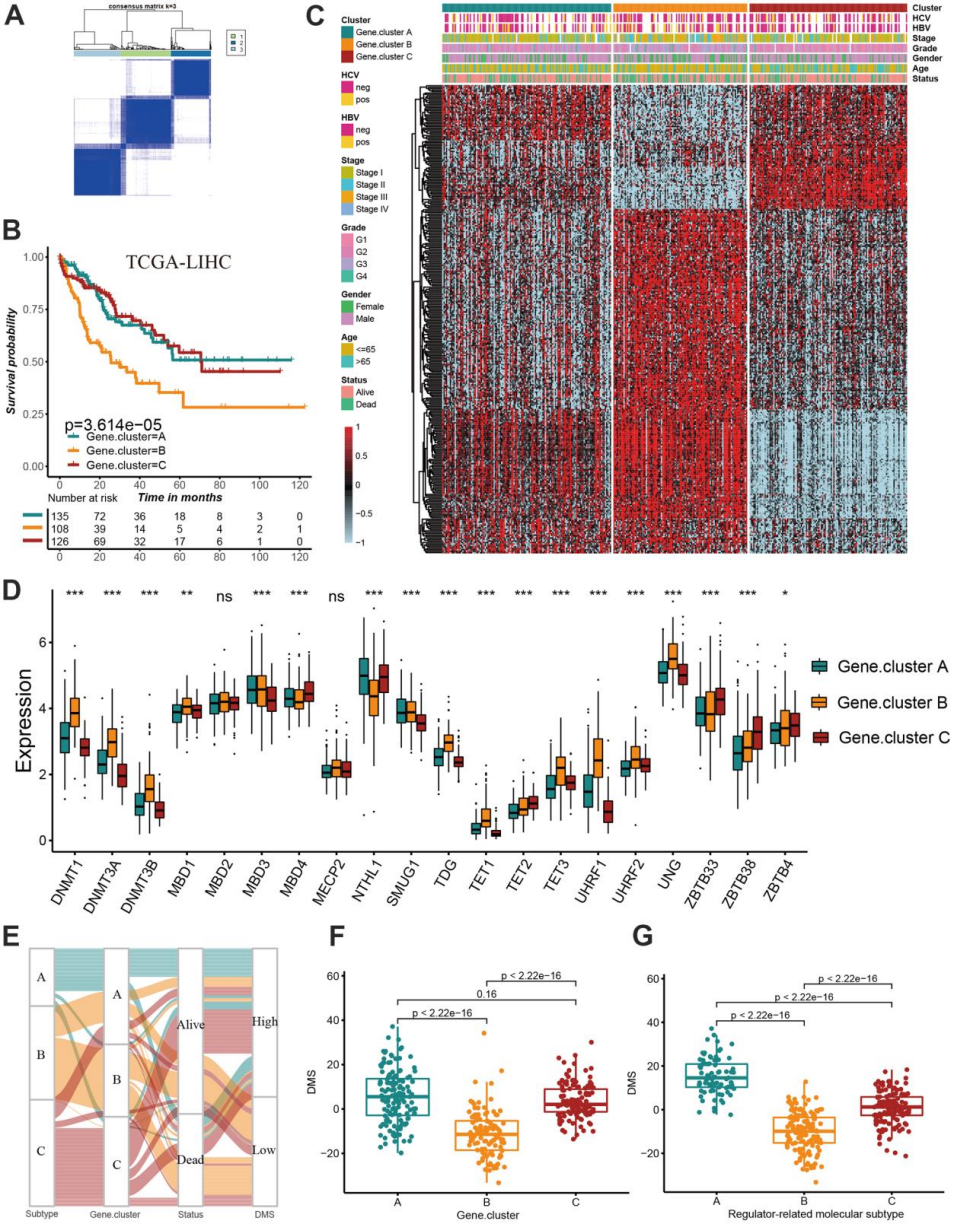
**Construction of prognosis-related DMS signature.** (**A**) Consensus matrices of DNA methylation subtype-related genes. for k=3. (**B**) Survival analyses for three distinct Gene.clusters. (**C**) The hierarchical clustering of1037 overlapping differentially expressed genes among three Gene.clusters. (**D**) Difference in the 20 DNA methylation regulator expression among three Gene.clusters. (**E**) The alluvial diagram showing the changes of sample attributes including methylation regulator-related molecular subtypes, Gene.cluster, survival status and DMS. (**F**) Differences in DMS score across three Gene.clusters. (**G**) Differences in DMS score across three distinct methylation regulator-related molecular subtypes. All data analyses were based on the TCGA-LIHC cohort. Neg, negative; Pos, positive; Ns, not significant. *P < 0.05; **P < 0.01; ***P < 0.001.

**Figure 5:**
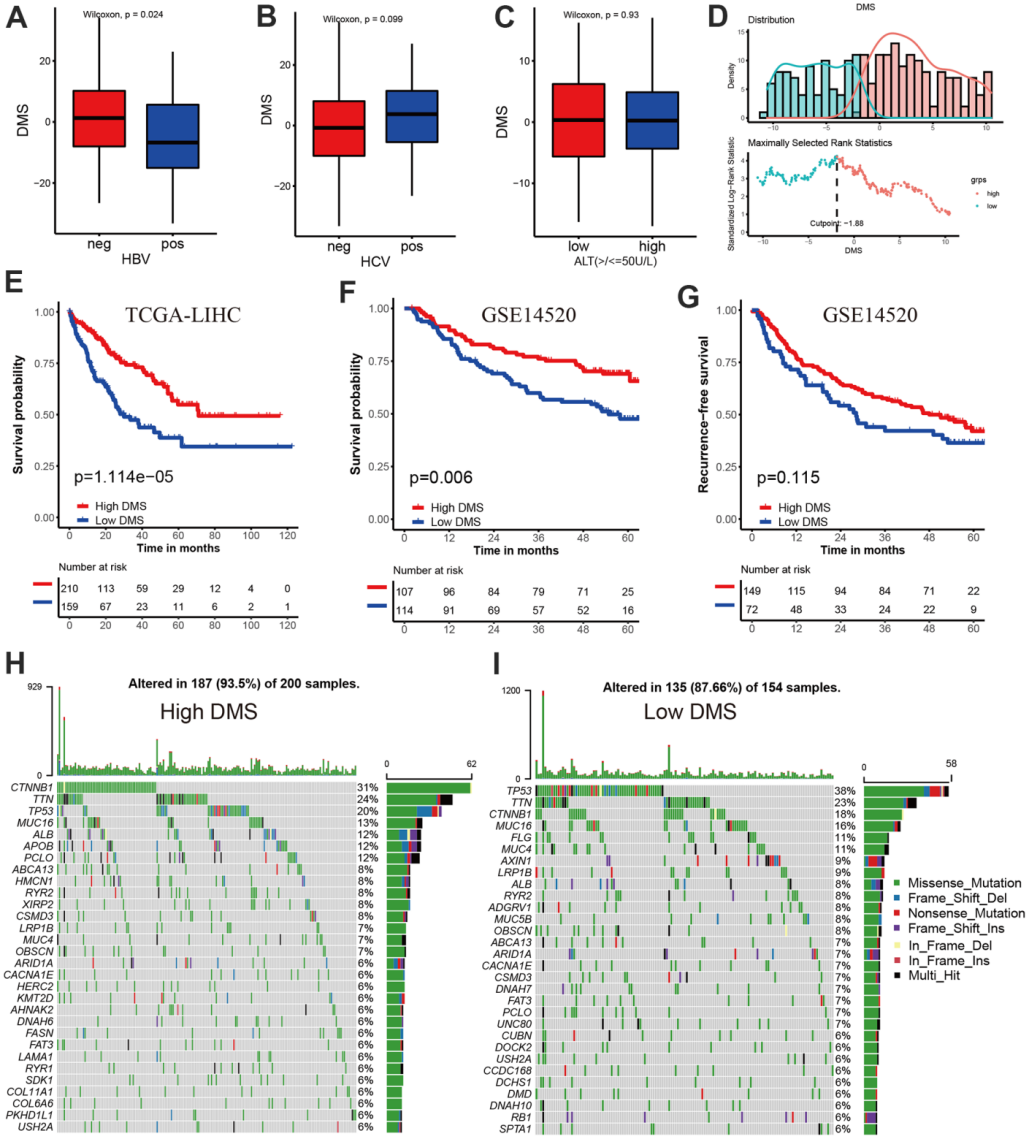
**Prognostic value and external validation of DMS signature.** (**A**) Differences in DMS score between hepatitis B virus positive and negative group in TCGA-LIHC cohort. (**B**) Differences in DMS score between hepatitis C virus positive and negative group in TCGA-LIHC cohort. (**C**) Differences in DMS score between ALT high and low group in GSE14520 cohort. (**D**) The MaxStat R package identified the optimal cut-off point to dichotomize DMS. (**E**) Kaplan-Meier curves showing the survival difference between the low and high DMS groups in TCGA-LIHC cohort. (**F**) External validation the value of DMS in predicting patient prognosis in GSE14520 cohort. (**G**) Kaplan-Meier curves showing the recurrence-free survival difference between the low and high DMS groups in GSE14520 cohort. (**H**, **I**) The waterfall plot showing the differences of TMB landscape between low and high DMS groups in TCGA-LIHC cohort. (**H**) High DMS group. (**I**) Low DMS group. Neg, negative; Pos, positive.

**Figure 6:**
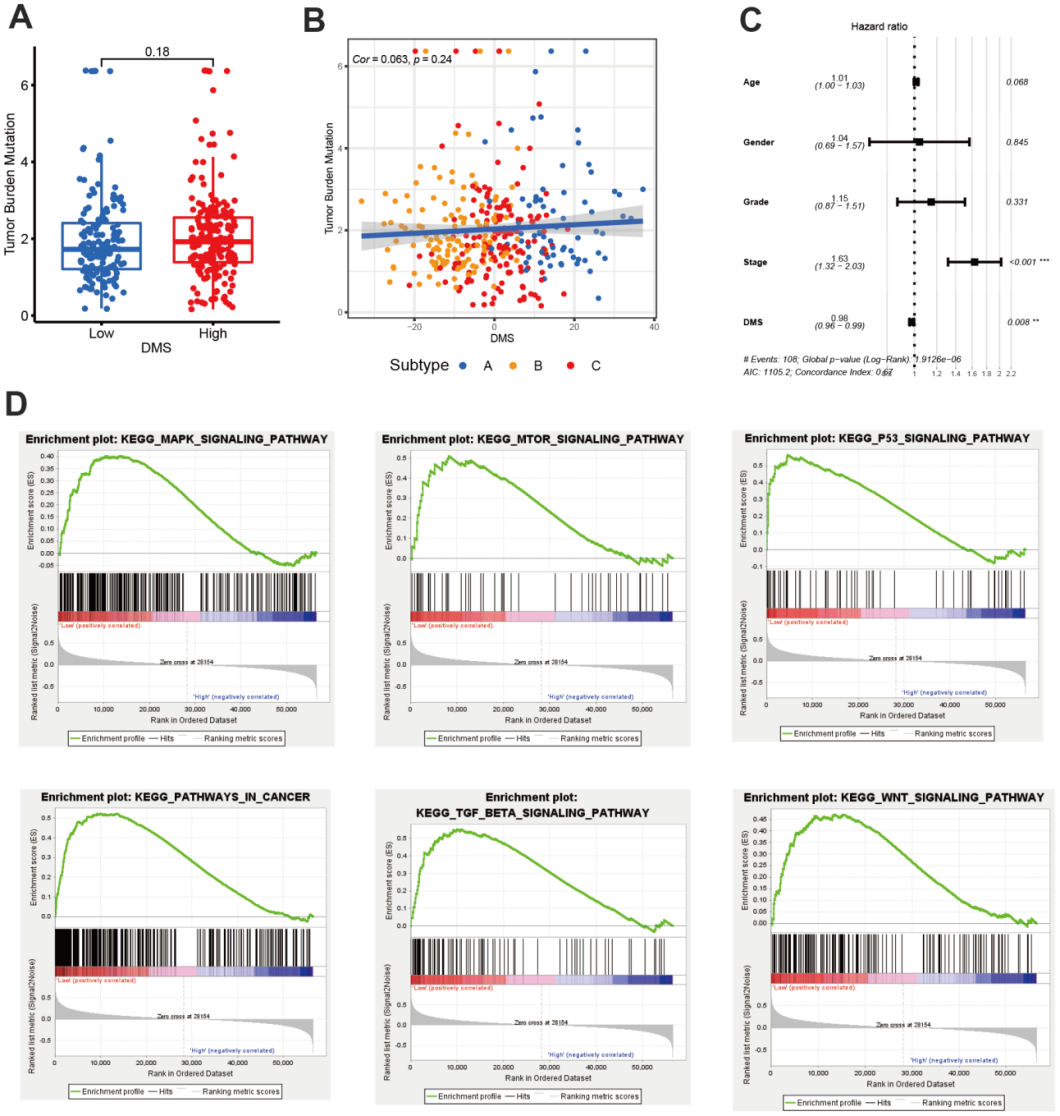
**The value of DMS in predicting clinical outcomes in patients with hepatocellular carcinoma.** (**A**) Differences in tumor burden mutation between low and high DMS groups. (**B**) The correlation between tumor burden mutation and DMS score. (**C**) Multivariate cox regression analysis for DMS in predicting patient’s survival in TCGA-LIHC cohort. (**D**) GSEA enrichment analysis showing the activated biological pathways in patients with low DMS. All data analyses were based on the TCGA-LIHC cohort. Cor, correlation.

We have showed that these 1037 genes were closely related to the three molecular subtypes we have identified. Therefore, we again performed consensus clustering on the expression of these 1037 genes to determine the ability to reproduce these three subtypes in hepatocellular carcinoma. The results confirmed that, based on the expression of 1037 genes, we could still identify three distinct subtypes in hepatocellular carcinoma, we termed these subtypes derived from these 1037 gene expression as Gene cluster A, B and C (Supplementary Figure 3 and Figure 4A). Survival analysis for these three groups showed a significant difference, prominent survival advantage in Gene.cluster A and C, and the survival inferiority in Gene.cluster B (Figure 4B). It was found the three gene clusters exhibited specific DNA methylation-related transcriptome characterization, respectively (Figure 4C). Further analyses indicated DNMT1, DNMT3A and DNMT3B showed a significantly high expression in Gene.cluster B compared to other two clusters, while ZBTB33, ZBTB38, ZBTB4 were remarkably up-regulated in Gene.cluster C (Figure 4D). Considering the crucial role of methylation regulator mediated molecular subtypes in prognosis, we constructed the prognostic signature based on prognosis-related overlapping DEGs using the PCA algorithm, which we termed as DMS signature (Supplementary Table 1). The alluvial diagram also revealed the changes of sample attributes including methylation regulator-related molecular subtypes, Gene.cluster, survival status and DMS (Figure 4E). We performed the correlation analysis between DMS score and 20 methylation regulator expression based on TCGA cohort, and found DMS score had the most significant positive correlation with NTHL1 (cor=0.58, Supplementary Figure 4), and the most significant negative correlation with DNMT1 (cor=-0.75, Supplementary Figure 4). Additionally, a significant distinction on DMS between methylation regulator-related molecular subtypes as well as between Gene.clusters was observed. Subtype A and Gene.cluster A displayed a highest median DMS, while the subtype B and Gene.cluster B displayed a lowest median DMS (Figure 4F, 4G). Hepatitis B virus positive patients presented a lower DMS (Figure 5A), while hepatitis C virus positive patients presented a higher DMS (Figure 5B). We did not observe the significant difference on DMS between low and high ALT patients (Figure 5C). Based on the optimal cut-point at -1.88 acquired from MaxStat algorithm, patients were classified as high and low DMS group (Figure 5D). Patients in the high DMS group experienced a remarkable survival benefit compared to low DMS group (Figure 5E). We then used the external validation set GSE14520 to validate the prognostic value of DMS in hepatocellular carcinoma. We still observed a significant survival advantage in high DMS group than low DMS group (Figure 5F). Although the statistical P value was insignificant, patients with high DMS still experienced an advantage trend in recurrence-free survival compared to those with low DMS. The landscape of somatic mutation in the low and high DMS groups was then further investigated. The tumor mutation burden did not show a significant difference across low and high DMS groups (Figures 5H, 5I, 6A, 6B). We used the waterfall plot to summarize the distinction on tumor mutation burden between low and high DMS groups (Figure 5H, 5I). We used the multivariate Cox regression model to further reveal the value of DMS in predicting patient prognosis, and found DMS signature was as an independent biomarker in predicting patient outcomes (Figure 6C). However, given the range of hazard ratio 95% CI (0.96 to 0.99, p = 0.008), we still needed to be cautious about this result. We than perform GSEA enrichment analysis, and found the classic carcinogenic signaling pathways were significantly activated in the low DMS group such as MAPK, MTOR, P53, TGF-beta and WNT signaling pathways, which could lead to the worse prognosis in patients with low DMS (Figure 6D).

### Role of DMS in predicting efficacy of immunotherapy

**Figure 7:**
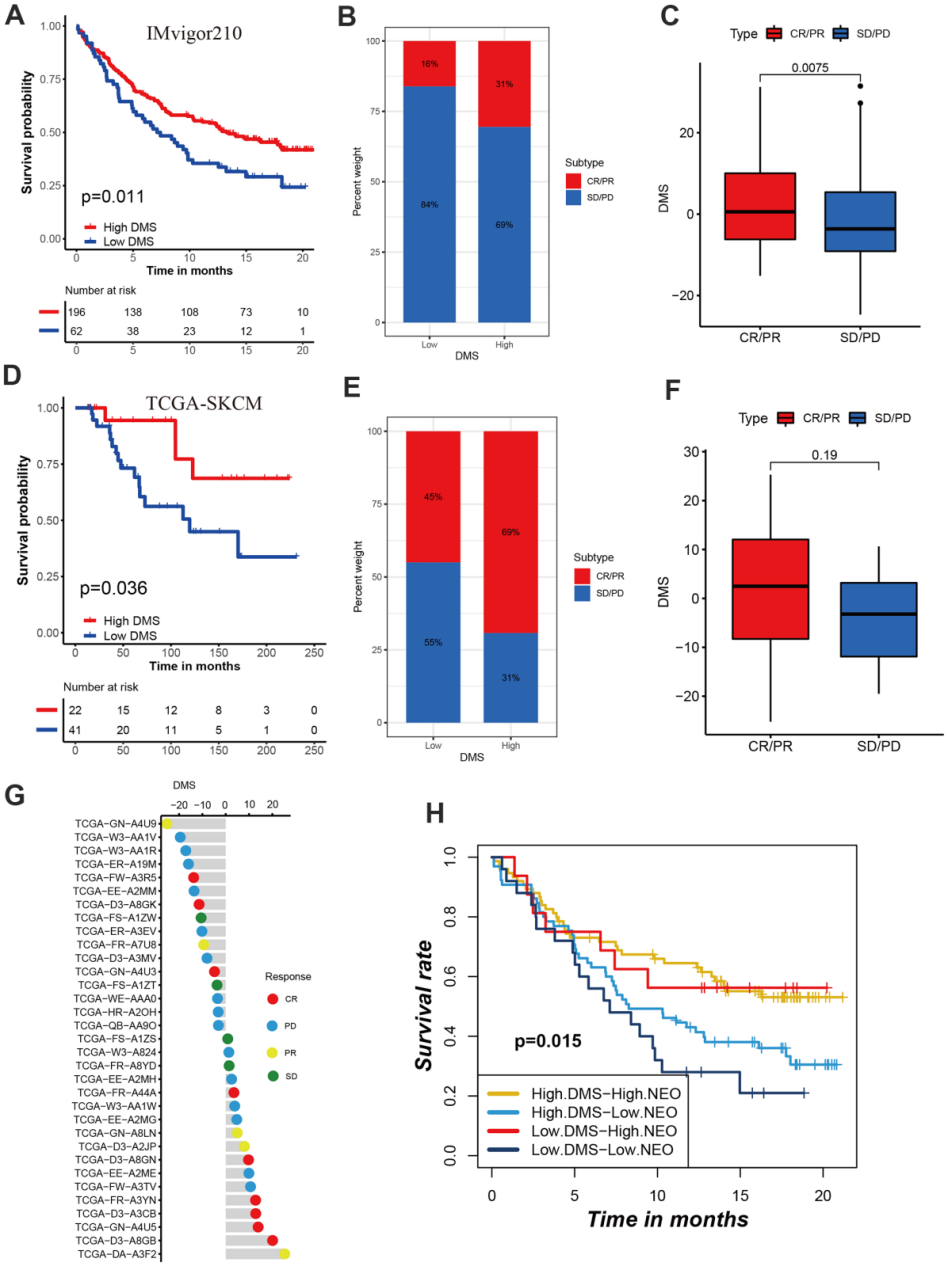
**Role of DMS in predicting efficacy of immunotherapy.** (**A**) Kaplan-Meier curves displaying the survival difference of high and low DMS groups in IMvigor210 cohort. (**B**) The ratio of clinical response types in high DMS and low DMS groups in the IMvigor210 cohort when treated with anti-PD-1 immunotherapy. (**C**) Differences in DMS score between different clinical response types in the IMvigor210 cohort. (**D**) Survival analyses for DMS in TCGA-SKCM cohort. (**E**) The ratio of clinical response types in each group in the TCGA-SKCM cohort. (**F**, **G**) Differences in DMS score between different clinical response types in the TCGA-SKCM cohort. (**H**) Survival analyses for patients receiving anti-PD-L1 immunotherapy stratified by both neoantigen burden and DMS signature. (**I**) Differences in DMS score among different immune phenotypes. CR, complete response; PR, partial response; SD, stable disease; PD, progressive disease. NEO, neoantigen burden.

The breakthrough of immunotherapy represented by immune checkpoints in cancer treatment has brought encouraging and instructive strategies for the successful cure of cancer. To further revealed the predictive values of DMS signature in patients treated with anti-checkpoint immunotherapy, we collected two immunotherapy cohorts with completed survival information including IMvigor210 cohort with the intervention of PD-L1 antibody and TCGA-SKCM cohort with the intervention of PD-1 and CTLA-4 antibody. In the IMvigor210 cohort, compared with patients with low DMS score, patients with high DMS score had a prominent clinical benefit and therapeutic advantage. The survival in the high-DMS group was significantly prolonged (Figure 7A–7C). In the TCGA-SKCM cohort, we still observed the treatment advantages in the high-DMS group than low-DMS group, emphasizing the predictive value of DMS signature in patient receiving immune checkpoint blockade therapy (Figure 7D–7G). In addition, when stratifying patients by DMS and neoantigen mutational burden, we found that patients with neoantigen burden as well as high DMS features had significantly improved survival when receiving immunotherapy (Figure 7H).

## DISCUSSION

With the in-depth understanding of the heterogeneity and complexity of tumor microenvironment, increasing evidence highlights the critical role of DNA methylation in inducing immune escape and immunotherapeutic drug resistance [15–18]. Nevertheless, DNA methylation regulator mediated molecular subtypes, microenvironmental characteristics under these subtypes as well as the impacts on immunotherapeutic efficacy still remain little known. Identifying the distinct molecular subtypes in order for classifying patients will promote the development of individualized treatment of cancer. Additionally, identification of the DNA methylation regulator mediated molecular subtypes may help reveal potential biomarkers significantly related to clinical response to immunotherapy and potentially uncover novel immunotherapeutic targets [19, 20].

In this research, we comprehensively integrated the transcriptome data of 20 DNA methylation regulators. Although all the regulators experienced a low genomic variation, there existed a remarkably difference in mRNA expression between normal and tumor tissues. According to the expression of 20 regulators, we successfully revealed three distinct DNA methylation regulator-related molecular subtypes in hepatocellular carcinoma, which had substantially different biological characteristics and prognosis. Subtype A was characterized by the higher expression of NTHL1, MBD3 and SMUG1, with a particularly prominent survival advantage; Subtype B characterized by the up-regulation of DNMT3A, TET1, DNMT3B, TET3, UHRF2, DNMT1, UHRF1and TDG, with a significantly survival inferiority; while ZBTB4, MBD4, ZBTB33, TET2 and ZBTB38 characteristically expressed in subtype C whose prognosis was similar with subtype A. The immune infiltration analysis for the three molecular subtypes showed the activated CD4 T cell and activated CD8 T cell presented a higher infiltration level in the subtype B whose survival was bad. We than further analyzed the characteristics of the biological function of each molecular subtype to clarify the reasons for the poor prognosis of subtype B with high levels of immune infiltration. We found numerous classic carcinogenic signaling pathways and stromal activity including WNT, p53 and TGF-beta signaling pathway were significantly activated in subtype B, which could result in a survival inferiority in subtype B. Subtype B exhibits characteristics of stromal activation, which may mediate immune escape of this subtype. Previous studies have classified tumors into three immune subtypes, including immune-inflamed, immune-excluded, and immune-desert. Although the immune-excluded subtype shows similar immune activation characteristics to the immune-inflamed subtype, the activated immune cells are localized around tumor cell nests without infiltrating into the tumor parenchyma, which leads to the false immune activation. Consistent with the characteristics of the immune-excluded subtype, immunotherapy can activate immune cells around the tumor, but cannot stimulate their infiltration into the tumor interior, resulting in a low response rate of such tumors to immunotherapy. [21–25]. TGF-beta promotes the growth, infiltration and metastasis of tumor cells by inducing immune escape, promoting blood vessel formation, and inducing epithelial-mesenchymal transition [26, 27]. In the previous studies, Zhang et al. also revealed three subtypes with distinct molecular, tumor microenvironment and clinical characterization in gastric cancer based on the expression of 21 m^6^A RNA methylation regulators, of these, the tumor microenvironment characterization under m6Acluster B subtype was highly consistent with immune-excluded phenotype [28]. Shen et al. reported three subtypes with distinct metabolic characteristics using the expression of 23 m^6^A RNA methylation regulators in hepatocellular carcinoma, and constructed m6Ascore signature to predict the prognosis and treatment response [29]. The above suggested that tumor heterogeneity could be further revealed by molecular classification of tumors by a specific gene set. At present, the transformation of cold tumors into hot tumors by targeting the immunophenotype of the tumor has become a hot topic in the field of cancer research. Previous studies have shown that MYC amplification mediated CCL23, CCL5, PD-L1, CD47 and IL1β expression decreased could induce macrophages and DCs inactivation, and also limit the recruitment of T cells, B cells and natural killer cells [30, 31]. Considering the high expression of DNMT3A, TET1, DNMT3B, TET3, UHRF2, DNMT1, UHRF1and TDG in Subtype B, changing the tumor microenvironment cell infiltration characteristics by reversing expression of these DNA methylation regulators may be more clinically practical. Based on the overlapping differentially expressed genes, we constructed DMS signature to further reveal the value of these molecular subtypes in evaluating patient’s prognosis and efficacy of immunotherapy. Subtype A exhibited a high DMS and subtype B showed a low DMS. DMS signature was proved to be an independent biomarker to predict the prognosis of patients. Additionally, DMS was correlated with hepatitis B and C. Similar to the subtype B, low DMS group was significantly enriched in the classic carcinogenic signaling pathways such as MAPK, MTOR, P53, TGF-beta and WNT signaling pathways. Using two anti-PD-1/L1 and anti-CTLA immunotherapeutic cohorts, we found the DMS signature could predict the patient response to immunotherapy. The checkpoint immunotherapy significantly improved the clinical response and prolonged the survival in patients with high DMS score compared with those with low DMS score. Generally, the high DMS groups improved more than 15% clinical response to immunotherapy than low DMS groups. This suggested that the DNA methylation regulator related gene signature could predict the efficacy and clinical responses in patients treated with anti-checkpoint immunotherapy.

## CONCLUSIONS

This study identified three DNA methylation regulator mediated subtypes with distinct clinical, molecular and biological characteristics in hepatocellular carcinoma, and constructed DMS signature, which could serve as an independent predictive biomarker in patient survival and response to immunotherapy. It may help promote individualized immunotherapy for hepatocellular carcinoma from the perspective DNA methylation regulators.

## MATERIALS AND METHODS

### Sample datasets collection and processing

In total, we collected 20 DNA methylation regulators based on existed published studies [12]. For training cohorts, we downloaded RNA sequencing data of TCGA-LIHC with FPKM types from The Cancer Genome Atlas (TCGA) Genomic Data Commons (GDC) Data Portal (https://portal.gdc.cancer.gov/) via TCGAbiolinks R package [32]. Then, we transformed the FPKM value into TPM values [33]. The copy number variation (CNV) data and somatic mutation were acquired from UCSC Xena public data hubs (http://xena.ucsc.edu/). The corresponding clinical information were curated from the TCGA GDC. The GSE14520 from Gene Expression Omnibus (GEO) database served as validation set [34]. The GSE14520, which was based on the Affymetrix Human Genome U133A 2.0 Array (GPL571), investigated the gene expression subtypes in tumor and paired non-tumor tissue of HCC patients as well as healthy donor liver. We used the affy R package to perform data preprocessing [35]. A total of 369 patients in TCGA-LIHC cohort and 221 patients in GSE14520 cohort with completed survival information were selected for further analysis. The median age was 59 years and 51 years in TCGA-LIHC and GSE14520 cohort, respectively. 255 patients and 170 patients were diagnosed with stage I/II in TCGA-LIHC and GSE14520 cohort, respectively. The baseline characteristics of patients in the TCGA-LIHC and GSE14520 cohorts was presented in Table 1. We included two immunotherapy cohorts after a systematical publicly search: The IMvigor210 cohort, which investigated the PD-L1 antibody in advanced urothelial cancer, was acquired from http://research-pub.Gene.com/imvigor300corebiologies. The raw count data was also transformed into TPM value. The TCGA-SKCM cohort, which investigated PD-1 and CTLA-4 antibody in advanced melanoma, was acquired from TCGA GDC data portal. The FPKM data was downloaded and then converted to TPM value.

**Table 1:** Baseline characteristics of patients in the TCGA-LIHC and GSE14520 cohorts.

**Cohorts**	**TCGA-LIHC (n=369)**	**GSE14520 (n=221)**
**Age, years**	59 (16 - 94)	51 (21 - 77)
**Sex**		
Male	248	191
Female	121	30
**Status**		
Alive	239	136
Dead	130	85
**Stage**		
Stage I/II	255	170
Stage III/ IV	90	49
Unknown	24	2
**Hepatitis B virus infection**		
Positive	44	212
Negative	147	6
Unknown	178	3

### Crosstalk among DNA methylation regulators

We constructed an expression-survival network to reveal a relationship of DNA methylation regulator connection, interactions and prognosis in hepatocellular carcinoma. We identified the protein-protein interactions (PPI) between DNA methylation regulators using the STRING database [36].

### Consensus clustering for twenty DNA methylation regulators

In order to identify distinct DNA methylation regulator mediated molecular subtypes in hepatocellular carcinoma, we performed consensus molecular clustering according to the mRNA expression of twenty DNA methylation regulators. The R package ConsensuClusterPlus was used and 1,000 times for subtype clustering were repeated in order for classification stability [37].

### Gene set variation analysis (GSVA)

The GSVA enrichment analysis was used to reveal the specified activated biological pathways among distinct DNA methylation subtypes. The enrichment score represented the relative activity of each biological pathway. The hallmarker and ‘c2.cp.kegg.v6.2.symbols’ get sets were acquired from the Molecular Signatures Database (MSigDB) for GSVA. We also estimated the tumor microenvironment abundance of immune cell infiltration including activated CD4 T cell, CD8 T cell, B cell and other cell types. The gene sets for estimating infiltration abundances were obtained from the published study [28, 38–40].

### Identification of differentially expressed genes and functional annotation

We identified the differences in mRNA transcriptome between the three molecular subtypes in hepatocellular carcinoma with the Limma R package [41]. The p value less than 0.001 was the criterion for screening the differentially expressed genes. Then we applied the R package clusterProfiler to perform the functional annotation for these differentially expressed genes [42]. The term BP (biological process) was selected for revealing the biological function of these genes.

### Construction of prognosis related DMS signature

First, we adopted the univariate Cox regression model to reveal the prognostic value for these differentially expressed genes in hepatocellular carcinoma. Then the principal component analysis (PCA) was performed for the expression of genes with the prognosis P value <0.05. The signature scores were composed of principal components 1 (PC1) and 2 (PC2) [28, 43]. The DMS signature was defined as follows:


$$\text{DMS}=\Sigma (\text{PC}1_{i}+\text{PC}2_{i})$$

where i is the expression of subtypes-related genes with a significant prognosis.

### Statistical analysis

The Kruskal-Wallis and One-way ANOVA tests was used to execute the difference significance test for three groups or more [44]. The difference analyses between the two groups was based on Wilcoxon test. The survival curves with the basis of log-rank tests and the Kaplan-Meier method were generated with the survminer R package. We classified patients into low and high DMS groups through the optimal cut-off point obtained from the MaxStat R package [45]. All statistical P-values were two-sided, and a p < 0.05 was statistically significant. All data was processed through the software R 4.0.5.

### Data availability statement

All data related to this work can be acquired from the Gene-Expression Omnibus (GEO, http://www.ncbi.nlm.nih.gov/geo) and the GDC portal (https://portal.gdc.cancer.gov/).

## Supplementary Material

Supplementary Figures

Supplementary Table 1
