# Supplementary material for: Molecular subtypes, prognostic and immunotherapeutic relevant gene signatures mediated by DNA methylation regulators in hepatocellular carcinoma
Source: Aging (Albany NY). 2022 Jun 30;14(12):5271–91. doi: 10.18632/aging.204155 (PMC9271297; doi:10.18632/aging.204155)
Supplement: Supplementary Figures [file aging-14-204155-s001.pdf]

SUPPLEMENTARY FIGURES

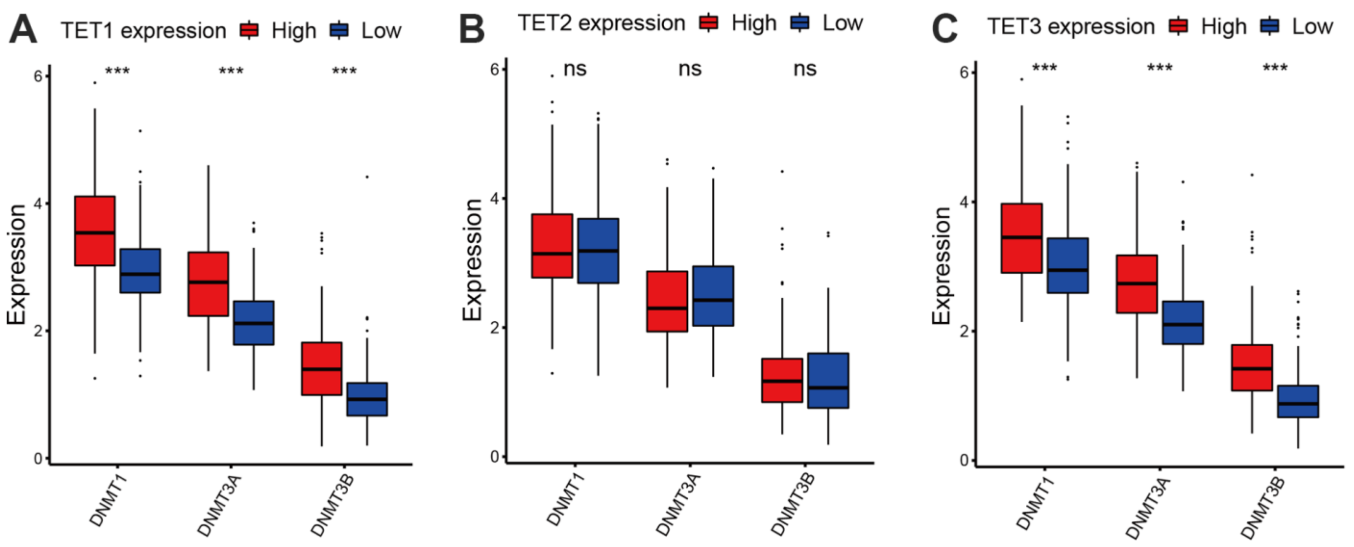

Supplementary Figure 1. Difference in writer gene expression between low and high eraser genes. (**A**) TET1. (**B**) TET2. (**C**) TET3. Data analyses were based on the TCGA-LIHC cohort.

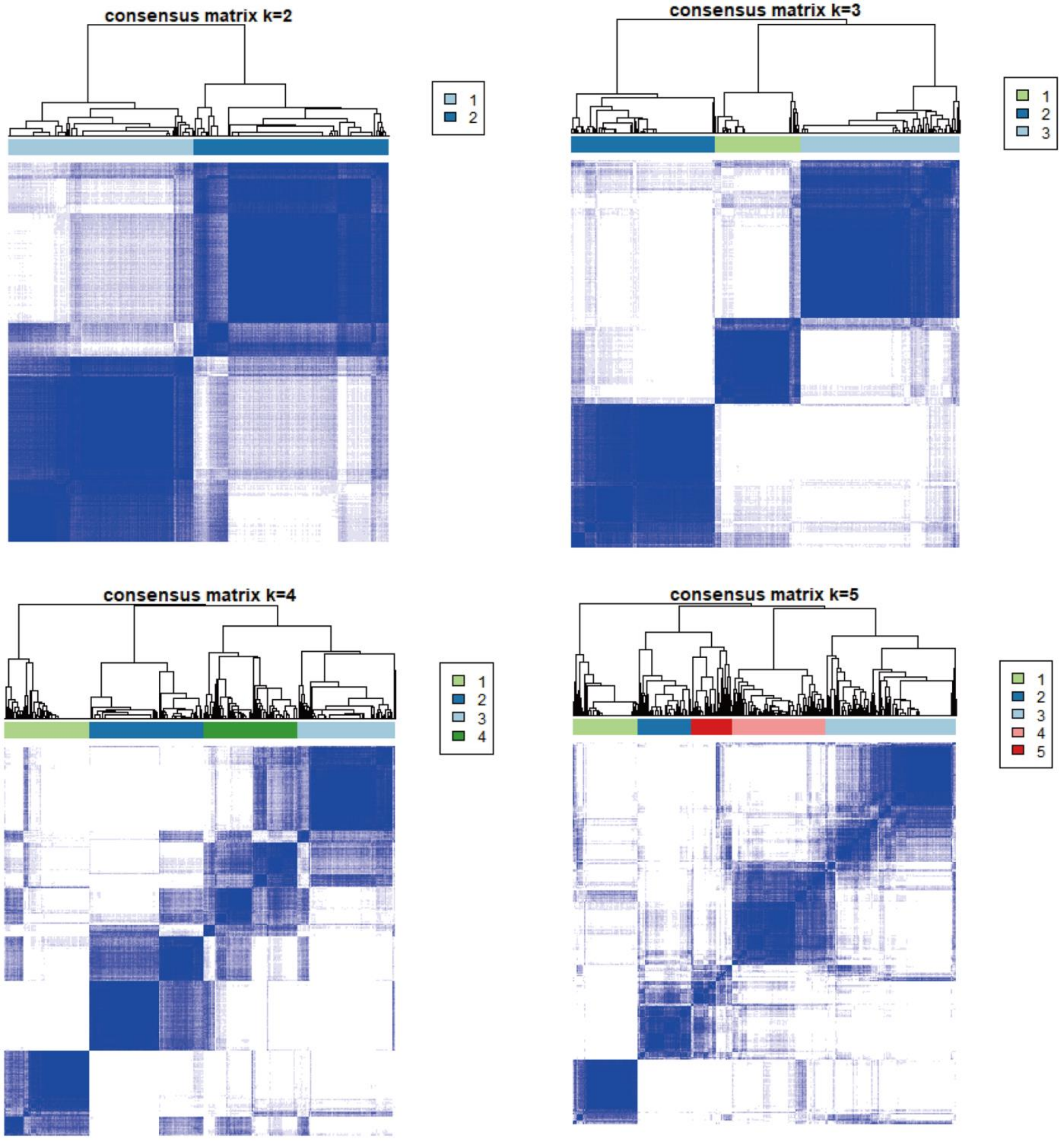

Supplementary Figure 2. Consensus matrices of 20 DNA methylation regulators for k = 2, k=3, k=4 and k=5. Data analyses were based on the TCGA-LIHC cohort.

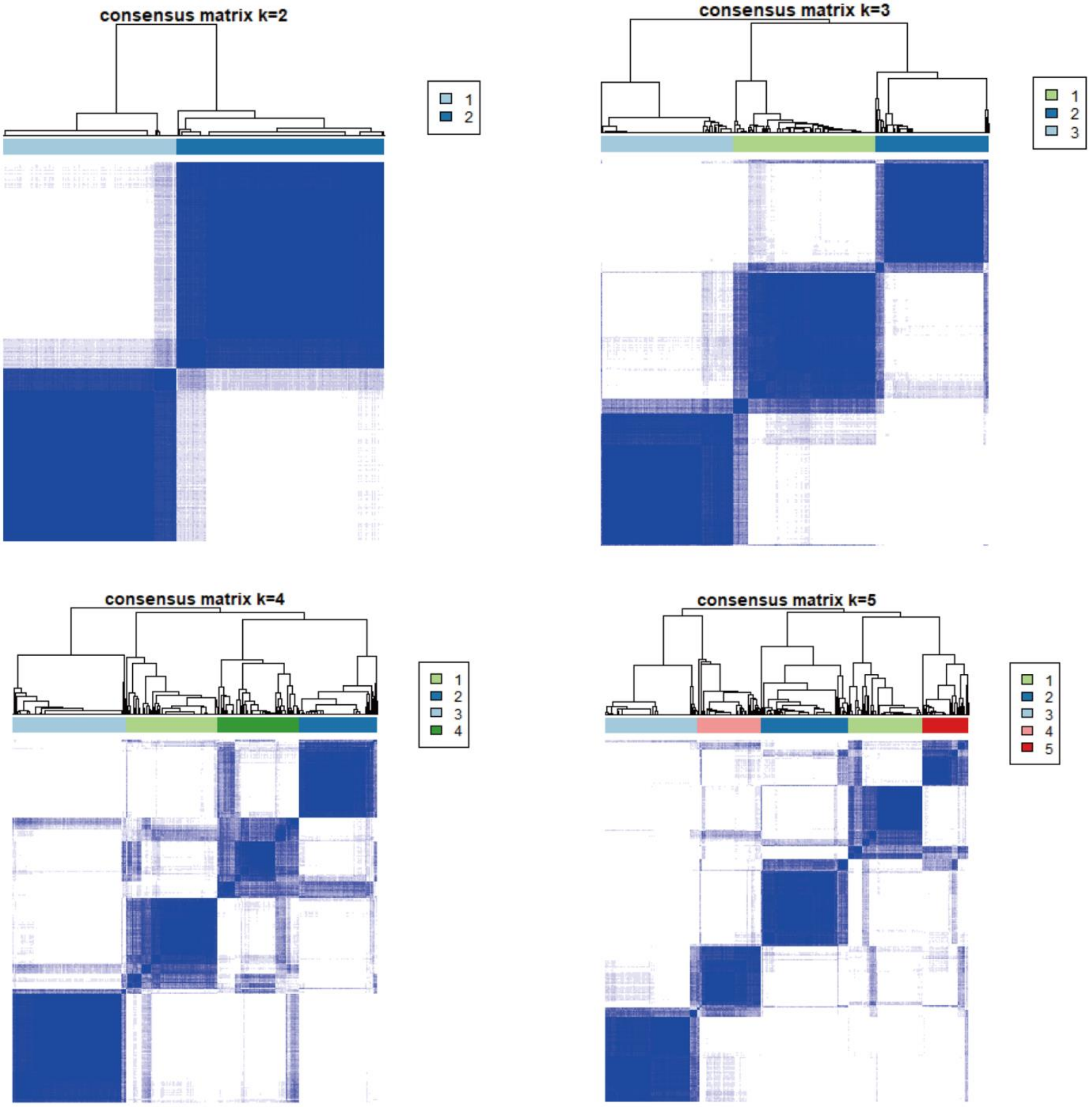

Supplementary Figure 3. Consensus matrices of 1037 overlapping differentially expressed genes for k = 2, k=3, k=4 and k=5. Data analyses were based on the TCGA-LIHC cohort.

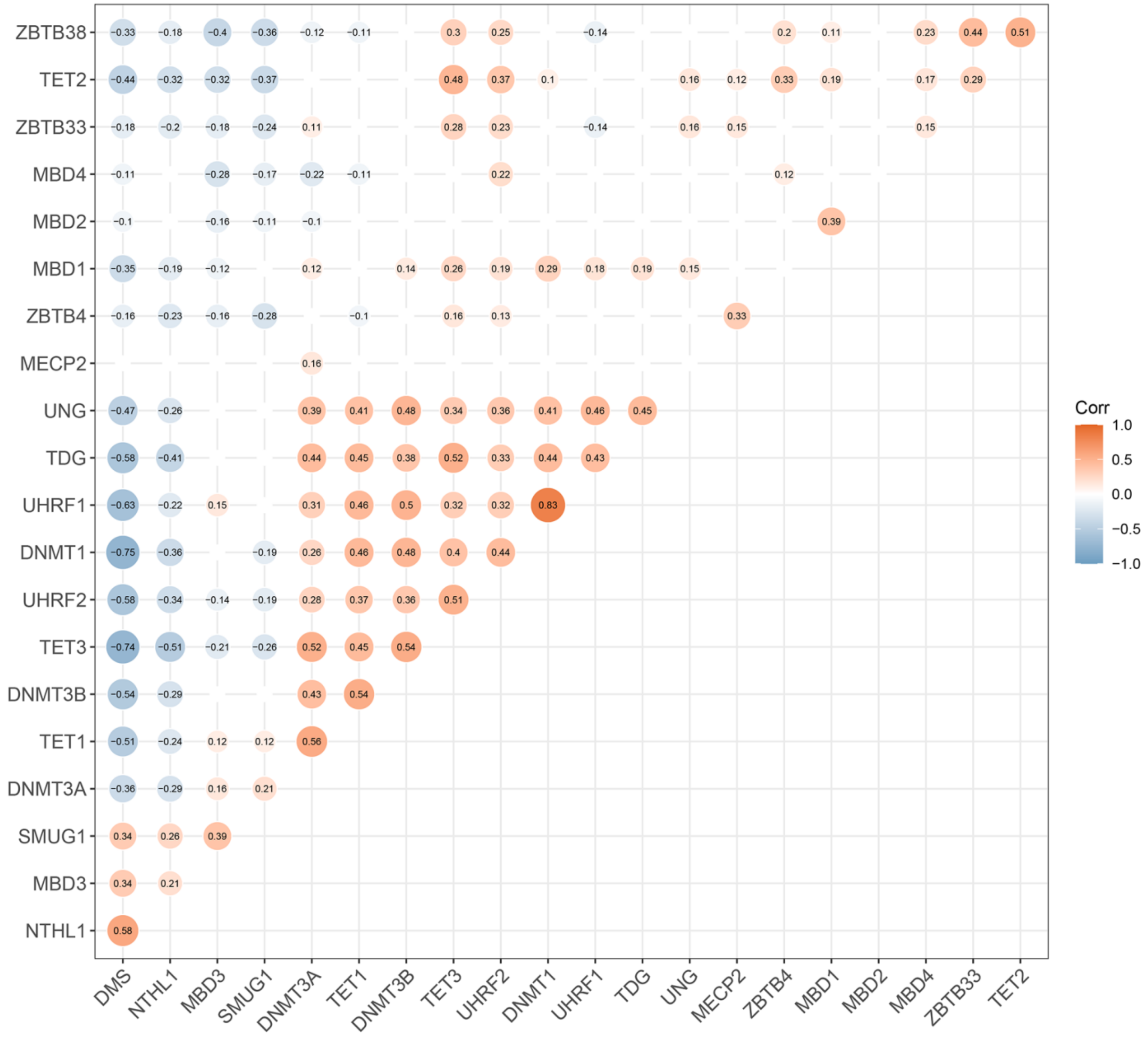

Supplementary Figure 4. Correlation between DMS score and expression of 20 DNA methylation regulators. The numbers in the circles represented the correlation coefficients.
